# Finding a safe space for learning and exploration: A qualitative study of recently diagnosed men’s experiences of peer support for HIV in Sweden

**DOI:** 10.1371/journal.pone.0283570

**Published:** 2023-03-30

**Authors:** Arielle N’Diaye, Tobias Herder, Anette Agardh

**Affiliations:** Division of Social Medicine and Global Health, Department of Clinical Sciences, Lund University, Malmö, Sweden; Sunway University, MALAYSIA

## Abstract

In Sweden men account for most new HIV cases, and little is known about the peer support needs of people living with HIV in Sweden. This qualitative study explored how recently diagnosed men perceive and experience peer support in Sweden. Purposively sampled from HIV patient organizations and infectious disease clinics throughout Sweden, data was collected through in-depth individual interviews with 10 men living with HIV, who have experience participating in peer support. Latent and manifest qualitative content analysis produced the overarching theme of *Finding a safe space for learning and exploration*. Participants used peer support to access key information and skills and as a space to safely explore life with HIV. Participants perceived successful peer support as having the right peer while also receiving support at the right location. Study recommendations include further research on how a peer is defined within the U = U era, further research on the peer support needs of young adults, and further research on the accessibility of peer support.

## Introduction

Peer support and its integration within health services is a form of social support that has been explored by many working in illness prevention and management for a variety of health conditions such as HIV, mental illness, and diabetes [1, 2]. For people living with HIV (PLHIV), previous study results suggest that peer support could be a key ancillary service to clinical care due to its potential to cultivate coping skills and assist with improving resilience [1, 3, 4]. Among recently diagnosed individuals in particular, peer support has been observed as being especially helpful with improving psychosocial outcomes and managing experiences of uncertainty [5, 6].

As a resource, peers provide support through exemplifying their experiences and giving advice that is rooted in previous actions [1, 7, 8]. A seminal and often referenced contribution, Dennis [8, 9] in her concept analysis on peer support in health care settings for PLHIV describes peer support as containing three components: emotional support (e.g., help with managing emotions), informational support (e.g., help with obtaining and understanding information), and appraisal support (e.g., help with assessing options and decision making). According to this conceptualization, peer support can be formal in the form of support groups, one-on-one support sessions, and mentorship programs organized by an organization [1, 5, 8, 9]. Peer support can also be informal in the form of casual support from family, friends, or romantic partners living with HIV [1, 8]. Additionally, within this conceptualization peer support is perceived as being bi-directional, where both support providers and recipients are positively, negatively, or neutrally affected by each encounter. This indicates that anyone experiencing peer support despite having the initial role of receiver or provider may potentially be influenced by engaging in support activities [1, 2, 5, 6, 8].

Peers can be defined in many ways and are usually defined by demographic group membership (e.g., women, senior citizens, men), behaviors (e.g., individuals in rehabilitation for substance abuse), or membership within an illness community (e.g., people with cardiovascular disease, diabetes, HIV, or cancer) [1]. However, in the literature about HIV-related peer support, peers are mostly defined as people who share the same health condition but not necessarily the same demographic or behavioral identifiers [1, 8, 9].

Since the 1980s there have been many developments in the field of antiretroviral therapy (ART), with approximately ten years having elapsed since the establishment of convincing evidence on infectiousness and the inability to sexually transmit HIV when virally suppressed [10–12]. With these advancements, not only has the life expectancy of PLHIV increased and is similar to those without the virus, but HIV has also become a manageable life-long chronic condition [11–14]. Despite this however, HIV remains highly stigmatized in many parts of the world [15]. Subsequently, peer support has shifted from primarily providing end of life support, to instead focusing on stigma management and supporting people to live long healthy lives [7, 16] which is reflected in the global U = U (undetectable equals untransmittable) campaign launched in 2016 by the Prevention Access Campaign [17].

In Sweden, it is estimated that approximately 8,000 people are living with HIV, with approximately 400–500 new cases each year [18]. In 2019 men accounted for 64% of new cases, with 38 years of age being the mean age at diagnosis [18]. Overall, Sweden has a relatively low HIV prevalence rate and in 2016 was the first country to officially achieve UNAIDS and WHO’s 90-90-90 targets [19]. This has been attributed to policies such as the Communicable Diseases Act, providing antiretrovirals free of cost, high levels of ART adherence, and using interdisciplinary care teams within specialty HIV clinics [20, 21]. Despite these efforts, little is known about the quality of life of PLHIV in Sweden, and even less in known about how PLHIV in Sweden use and experience peer support to meet needs that fall outside the sphere of the medical and social service system [19, 22].

As such, due to the widespread use of ART and subsequent viral suppression, questions can be raised about the role of peer support at a time when PLHIV can expect similar life trajectories as people not living with HIV. Thus, this qualitative study aimed to understand the meaning of peer support for recently diagnosed men living with HIV in Sweden, and whether peer support for PLHIV is still needed. Our main research question was *how do recently diagnosed men living with HIV in Sweden perceive and experience peer support*? This study is situated within what we have called “the U = U era”, where participants have experienced adjusting to life with HIV during a time when scientific evidence concerning the effectiveness of ART became increasingly known. Additionally, we defined peers similarly to that of other studies on peer support for PLHIV, where peers are defined as individuals who share the same health condition of living with HIV but not necessarily the same demographic or behavioral identifiers [1, 8, 9].

## Methods and materials

### Participant criteria

Study participants were recruited through purposive sampling from March 2020 to November 2020. This study had the inclusion criteria of self-identifying as male, being at least 18 years of age, having lived with HIV for at least two years and no more than ten years, and having experienced peer support (either giving or receiving). The inclusion criterion of having lived with HIV for a minimum of two years and a maximum of ten years was selected to explore the experiences of men adjusting to life with HIV within the U = U era. The inclusion criterion of either having provided or received peer support was chosen because existing literature suggests that peer support could be a bi-directional experience within both formal and informal contexts [1, 2, 5, 6, 8]. Therefore, it was decided that including this criterion was essential to obtaining nuanced answers to this study’s main research question.

### Sampling strategy

Participants were purposively recruited through patient organizations and infectious disease clinics. At a partner patient organization for people living with HIV, organization staff disseminated information about this study online on their social media channels and within their network of other HIV patient organizations. At infectious disease clinics, clinic staff assisted in recruitment by displaying promotional posters in clinic waiting rooms and by providing promotional flyers to potential participants during their appointments. Both the posters and the flyers provided details about whom on the research team prospective participants should contact if they were interested in participating. After contacting a member of the research team (AN), participants were provided with an information letter.

After receiving the information letter and indicating that they were still interested in participating in the study, participants then arranged a time for their interview with a member of the research team (AN). Before beginning each interview, participants read through the information letter and consent form before confirming again that they would like to participate in the study. This recruitment strategy yielded 10 participants. Recruitment ended after it was established that sufficient information power had been reached [23]. This occurred through having a specific study aim and a clearly defined study population that brought forth interviews yielding rich and nuanced conversations.

### Data collection and analysis

Prior to beginning the data collection process, the research team consulted with staff at a partner HIV patient organization to understand the dynamics at play when interviewing PLHIV within a research study context. Data was collected through individual in-depth interviews, using a semi-structured interview guide [24]. Interviews ranged between 50 and 65 minutes in length. This interview guide was divided into three sections: *What is peer support*? *Who is a peer*? *How do men access*, *navigate*, *and participate in peer support*? Interviews were conducted in person at a patient organization (2 participants) and over video call (8 participants). Interviews were conducted in English by the first author (AN), who is fluent in English. A Swedish language interview option with research team members fluent in Swedish (AA, TH) was provided if participants preferred to be interviewed in Swedish. All participants chose to be interviewed in English. Zoom (version 5.0.4) was used for interviews by video. Conducting interviews over Zoom was decided to be a viable option because studies have shown that it allows for a comparable experience to that of in-person interviews [25, 26]. All participants consented to their interviews being audio recorded. All interviews were audio recorded and transcribed verbatim.

Transcripts were analyzed using manifest and latent qualitative content analysis with an inductive approach [27–29]. Individual interviews were the unit of analysis. From the transcriptions, meaning units in the form of sentences and paragraphs were condensed and labeled with a series of codes. Codes were grouped into subcategories and then aggregated into categories. Finally, categories were examined for the development of themes. Analysis was initially completed by one member of the research team (AN). Following this, the analytical matrix was examined by the other co-authors (AA, TH) to obtain feedback and consensus on the findings. Throughout the analysis and data collection processes, a reflexive journal was kept by AN. The contents of this journal were discussed with members of the research team while obtaining consensus on study findings to minimize instances of bias. After consensus was reached, member checks were used to increase the confirmability of study results. This study is reported in accordance with the Standards for Reporting Qualitative Research (SRQR) [30].

### Ethical considerations

To protect the privacy of participants and maintain their anonymity, specific measures were taken. In-person interviews were conducted in a private room where participants could not be overheard, and participants chose to be interviewed at their local patient organization. Prior to being interviewed over Zoom, participants were asked to select a space that was comfortable for them and maintained their privacy as well. The member of the research team conducting the zoom interview was also in a room where they could not be overheard. Before beginning each interview, each participant received a participant ID and was instructed to use this ID instead of their names when contacting the research team with any questions. After each interview all email communications between study participants and members of the research team were deleted. During analysis, interview transcripts and meaning units were anonymized by removing any identifying information. All transcripts and audio recordings were kept on an encrypted disk and in a locked cabinet accessible only to research team members. No renumeration was provided to participants. In response to the situation surrounding COVID-19 the research team had to consider the ethical implications of traveling to interview participants, and subsequently decided to provide participants with a video call option.

The inclusion criterion of living with HIV for at least two years was decided after consulting with staff at a partner patient organization for PLHIV. From these consultations, it was decided that potential participants needed at least two years to begin adjusting to life with HIV before an interview could be conducted without an increased risk of harm being done. However, in the event that difficult emotions arose during interviews, participants were given the opportunity at the end of each interview to debrief. While debriefing, participants were able to ask questions and discuss topics related to the study. Moreover, after each interview participants were also provided a resource list with the contact details of HIV patient organizations and health centers they could reach out to as well. This study has approval from the Swedish Ethical Review Authority (Dnr 2019–06511) and was done in accordance with the ethical standards specified in the Declaration of Helsinki [31].

## Results

10 men between 25 and 47 years of age were interviewed, with a mean age of 33 years old. The mean period of living with HIV was 7 years. The number of years participants had been living with HIV was as follows: 2 years (N = 1); 3 years (N = 1); 6 years (N = 2); 7 years (N = 2); 10 years (N = 4). Most participants resided in urban locations (N = 8) and two participants resided in rural locations. Most participants identified as gay (N = 6), three identified as heterosexual, and one identified as bisexual. The majority of participants were raised in Sweden (N = 8), while two participants were raised in countries outside of Sweden.

**Fig 1:**
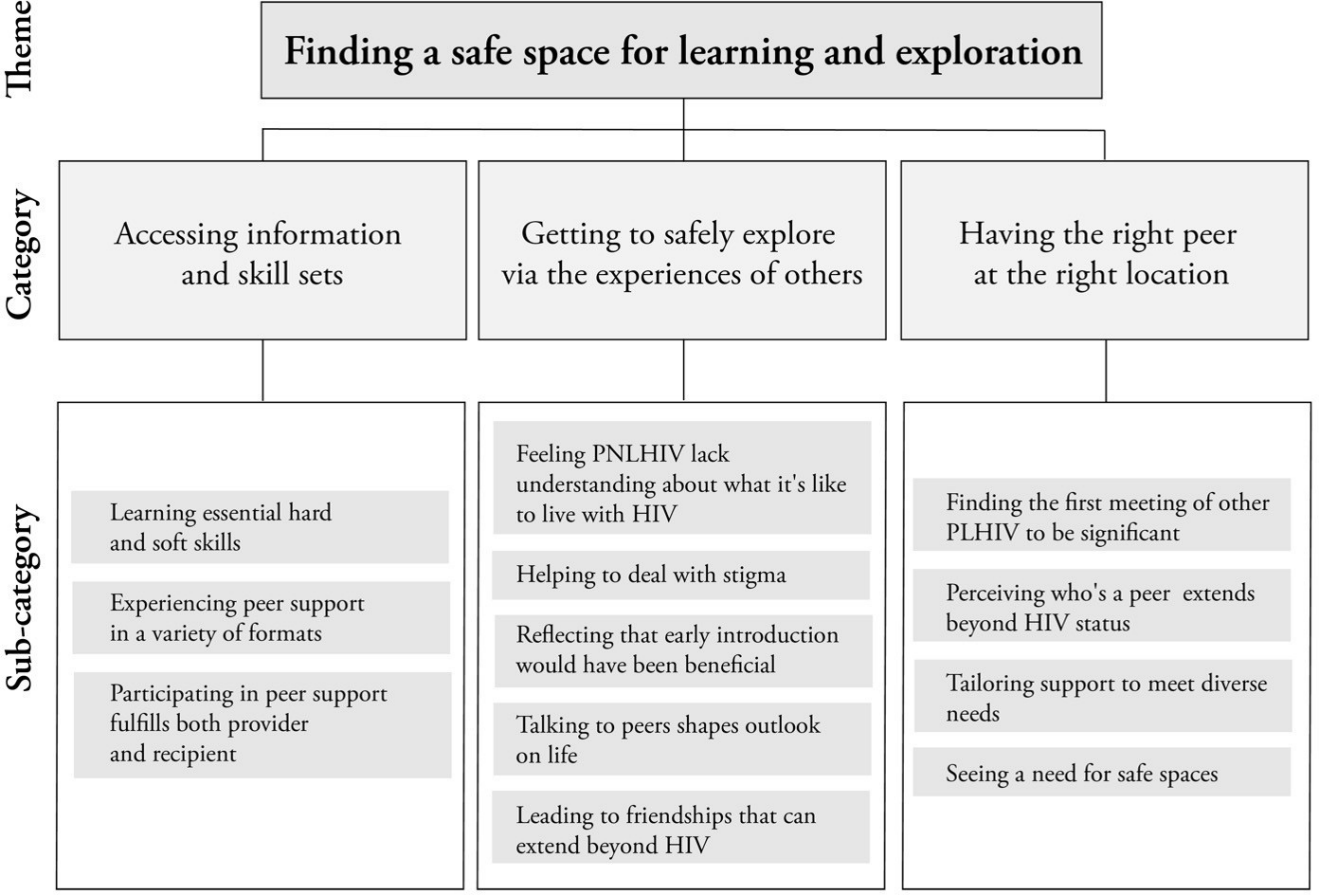
Summary of analytical model.

Based on the analysis of these interviews, the emerging theme of *Finding a safe space for learning and exploration* was identified. This theme was derived from 12 sub-categories and 3 categories (Fig 1). These categories—*Accessing information and skill sets*, *Getting to safely explore via the experiences of others*, and *Having the right peer at the right location—*are further described below.

### Accessing information and skill sets

Prior to diagnosis, the majority of participants expressed having limited knowledge about HIV. When diagnosed, they reported not knowing that HIV functions like a chronic condition when managed with medication.

When I was diagnosed already at the hospital, when they told me and I asked how long I was gonna live, and then they were like “Woah. Don’t you know anything?” And I was like “No.” (P4).

Because of this limited knowledge, in the period shortly following diagnosis some participants mentioned feeling panicked after missing a dose of their medication or after losing their medication. This fear was rooted in not knowing the appropriate actions to take when a dose is missed.

[While on a trip shortly after receiving the diagnosis] my luggage was gone with my medication in it for two weeks, and I was horrified because I thought I was going to die and I didn’t know anything and [my family] didn’t know anything, and I was so upset the whole trip (P7).

Because of this, peer support played a critical role in providing participants with information about the infection, and opportunities to workshop the skills needed to live successfully with it.

When engaging in peer support, many used it as a space to learn essential hard skills. Some of these skills included learning where to seek practical information about HIV, successful strategies for status disclosure, and understanding the mechanics of the infection. Moreover, participants also attended peer support to gain key soft skills. Some of these skills included learning how to get to know other PLHIV and learning the strategies that others have used to emotionally adapt to life with HIV. When learning these new skill sets, participants perceived peer support as facilitating their ability to talk about topics that intersect with life with HIV—such as dating, coping strategies, and opportunities to meet other PLHIV. Participants acquired these skill sets and information through participating in a variety of peer support formats. Some participants perceived peer support as something that was only provided within a formal context at a patient organization. Yet despite this perception, participants experienced both giving and receiving peer support in a variety of ways. Some of these included formal avenues such as activities and support groups organized by organizations, and informal avenues such as engaging with partners, friends, and acquaintances.

Yeah, I think it can happen in any kind of way, in any kind of formation. It can be friends over coffee, over lunch, during a walk, it can be more … I don’t know professional… it can be any way I think (P7).

Participants (N = 6) who provided peer support in a formal capacity did so while holding positions (paid and unpaid) within HIV organizations. The transference of skills and information was bi-directional, where peer support providers also acquired new skills as well. Providers experienced providing peer support as something that gave them confidence. They also experienced it as a pleasurable experience that gave them a sense of fulfillment and gratification.

It’s very gratifying and it doesn’t have to do with ego gratification you know what I mean? […] So something happens that I stop being me a little bit. It’s not about me when I’m there… you know? Which is quite interesting because you know there’s of course a part of me that likes to be there and I like to give help, and I know I’m doing a good thing (P6).

For some, this gratification stemmed from being able to help others in a way that they were once helped.

Participants gave peer support for a variety of reasons. Some were motivated to provide peer support out of a desire to change how PLHIV lived with HIV. Others did so because they saw how a lack of access to information about HIV resulted in negative health outcomes among those they knew.

I just knew that probably that something… this should be better […] Like living with HIV, or with how you deal with it […]. And there has to be something I can do, and [providing peer support] was probably the only way to like do something more actively […] (P1).

My motivation of talking to people is back in Africa. I’ve seen people out of negligence and stupidity and ignorance pass away. I know a couple of people. Lots of people pass away because they don’t want to be seen in the clinic, they don’t want to be taking pills (P10).

Others provided support with the goal of instilling confidence and the ability to do difficult things. During this process, some participants tried to push recipients to achieve key skill sets and milestones which they themselves might not have accomplished yet. This two-way exchange of benefits contributed to a continued learning of skills for peer support providers.

If I have given peer support, I will give something that strengthens you in any case. I will try to understand you, I will let you vent, but at the end of the day I will want you to go out of here feeling a little bit taller (P1).

### Getting to safely explore via the experiences of others

As described by the participants, peer support served as an opportunity to explore what it is like to live with HIV via engaging with the lived experiences of others. In doing so, participants were able to learn from both positive and negative experiences without having to go through these experiences themselves. Participants explored experiences such as stigma management and others’ regret of not having accessed peer support while adjusting to life with HIV. For participants, having a space to safely discuss their fears, to share negative experiences, and to see the resilience of other PLHIV was important. It resulted in them gaining a positive outlook on life, and forming friendships that eventually progressed beyond providing HIV related support.

When interacting with people not living with HIV (PNLHIV), many participants perceived them as not understanding what coping entails for PLHIV. Moreover, PNLHIV were viewed as not recognizing that having HIV was stigmatized by society at large.

There’s still like a great ignorance in the society, so like some people would even think […] that they are going to die within the next weeks or months or so… [HIV] is still like a chronic disease that you will be living with for the rest of your life so that in itself is also something that could be very difficult to cope with (P3).

And now we think that we’ve normalized it so now it’s not an issue at all and you don’t have to deal with telling people and you don’t have to deal with stigma within the society or people still being scared of you […].We don’t talk about people’s feelings or narrative, or how it impacts people’s lives, or how you feel or how you react to it, or how you cope with it (P2).

Additionally, some participants experienced the support from other PLHIV as carrying more weight and validity than support from PNLHIV sources. This was attributed to PLHIV having the experience of living with HIV and helping participants to humanize the infection.

There is a thing with authority, not that [a specific patient organization]is an authority, but it’s more like an authority. When they say something, if they say the exact same thing [that] my dad tells me, everything is fine blah blah blah, it weighs more (P9).

Among participants who did not seek peer support, some expressed receiving support from PNLHIV sources such as friends, family, and partners. These participants voiced using PNLHIV sources as spaces to safely explore their questions and anxieties.

So, then I started to get to the psychologist and stuff. And then I never sought peer support for HIV because I was offered support for my PTSD and stuff, so I thought it was almost involved in this kind of support I already have (P7).

I had a big support from my family, from my parents, and from friends, so I didn’t feel the need to reach out to anyone (P9).

Participants expressed a fear of being stigmatized, especially after disclosing their HIV status to others. As a result, participants sought peer support because they perceived it as something that would give them the strategies needed to deal with stigmatization. They found stigma management to be an especially challenging task.

I think it’s extremely [needed]. Peer support, because it has nothing to do with you cannot transmit the virus or not. You need peer support because of stigma in the society. That is why you need peer support. Because people are afraid of HIV, and people are afraid of people living with HIV because the knowledge level is so low […] We still need peer support because of the stigma in the society (P4).

When reflecting on their experiences of learning to live with HIV, participants voiced that having an early introduction to peer support would have been beneficial. They spoke of how early access to peer support promotes adopting positive behaviors, while also shortening the journey from a moment of crisis to a state of adaptation.

Peer support quickens the healing process […] I would have found my way here by my own, but it shortened the leap from getting the diagnosis to today. So, it quickens the way when you have someone that has lived through something a bit life changing (P5).

Those who did not participate in formal peer support early expressed regret and felt that participating in peer support would have made the adaptation process easier for them. Specifically, they viewed peer support’s ability to provide a non-judgmental and risk-free space to ask questions and learn from others as something that would have helped them to better navigate the adaptation process.

[Looking back] I’d tell myself to take every support I could take. Because it would make it easier to go through things together with people who might go through the same thing, although we haven’t the same experience in life. But I think yeah, I would tell myself to take more support. Take because it’s free and there for me, and no one will judge you. It’s stupid not to take it … not stupid, but yeah (P7).

Furthermore, all participants expressed the view that peer support is a service that is still needed for recently diagnosed individuals and should be offered to everyone. They also perceived that peer support should be voluntary.

It should be optional to get peer support and it should be something that should be provided to the people that want it. I think that it could be potentially harmful to provide it as something that’s obligatory […]. But it’s something that is good to be able to provide for people who desire it (P3).

Participants described how talking to peers was instrumental in shaping their outlook on life. For many, the first person they contacted after diagnosis was another PLHIV. This was because speaking to peers helped participants to comprehend and conceptualize a life with HIV. They found that the words and experiences of those providing peer support helped them to change inaccurate and negative perceptions.

I went to the meetings and […] and that was the first people I met, and it changed my way… how I looked upon it totally. [HIV] could be anyone […] we could look anyway, we could be black, we could be white, we could be heterosexual, homosexual, male, female, whatever; old, young, they were everybody. And so I learned that we’re just people (P4).

I think the most positive thing is maybe that you don’t feel alone. And I enjoy the diversity of the people sharing this thing. Because when you think about [HIV], you don’t think about [PLHIV] as being a diverse group with different personalities (P9).

Additionally, participation in formal forms of peer support for many participants led to the development of friendships with other PLHIV.

I also talked to my friends at [the patient organization]. How they were dealing with relationships, and comparing myself to them, how did they… some of them had relationships, some of them didn’t (P4).

For some, friendships with other PLHIV constituted their most central form of peer support and developed to a point of relating to each other beyond the basis of HIV.

He was also the one telling me I gave him peer support in the way that I was open and so you know, didn’t really care about [having] my HIV status out. He had an issue with that, and he had an issue with a lot of other things. But he could also correct me in many ways, and I could correct him [too]. I would teach him or help him in other ways so that was, and we’re still very good friends […]. So, he continued to be that solid rock and this special friend. That is now not the same, we don’t talk about HIV at all when we meet today […] (P2).

### Having the right peer at the right location

Participants experienced positive peer support experiences as consisting of two components: 1) engaging with the right peer, and 2) receiving support at the right location.

Participants described finding the “right” peer as a key component of a successful peer support experience. This was because peers are responsible for providing not only information, but also emotional support and a space to share personal experiences. According to participants, the person whom they regarded as a peer could vary widely. A peer could be a role model, could be any age, and could share similar characteristics or experiences in addition to having HIV. For those who had a partner also living with HIV, they found comfort in having a partner that was also a peer. This comfort arose from having someone with an implicit understanding of what it is like to live with HIV and having someone who exemplifies that it is possible to live well while also having HIV.

I remember my boyfriend telling me [after receiving my diagnosis] “It’s fine. It’s gonna be fine. It’s gonna be super fine”. Like very you know, like no deep talk about anything, but I just knew that it was (P6).

[…] I would never choose someone with HIV much more than someone without HIV… I don’t care about it. It just happened. I just found it actually easier when he actually has it (P7).

Both before and while attending their first peer support meeting, participants described feeling a wide array of emotions. These emotions ranged from feeling curious, exciteted, and welcome; to feeling nervous and fearful. The root of these feelings was diverse in nature. For participants with an immigrant background, these feelings sometimes came from previous experiences of judgement and stigmatization that had occurred in their home countries.

The first time I was scared [there] would be this hard African mentality, the African feelings that people are going to look at me, and say things, and put me in a group, or say something negative about me…. see all my flaws…and probably people who are [in the building of the patient organization], people around there will stigmatize me (P10).

I still remember the first time we met, shook hands, and I kind of just looked at him and said like so this is what a person with HIV looks like […]. But it was a nice relief (P1).

Interestingly, some participants also viewed PNLHIV who had a close proximity to HIV and chronic disease as peers. Such individuals could be patient organization staff, family, friends, and medical staff. This was because these individuals were seen as being able to empathize and understand what PLHIV were going through.

[…] he smoked a lot and drank wine and had chronic pain … so he knew about pain, and living, and enduring it (P5).

But he’s told me about his, like he has this depression and all that, so I figured yeah, I’ll tell him about this (P8).

Moreover, having the right peer was seen as important because participants experienced the first meeting with another PLHIV as a significant and important moment. In a similar vein, participants felt that finding the right peer included being provided with support that could be tailored to address identity-specific needs if needed. They expressed that an individual’s peer support needs might differ depending on demographic factors such as their geographic location, race, ethnicity, age, sexual orientation, and nationality.

[…] but I also think that it is minority stress […], so that can impact more people than just people with HIV. For example, [among] gay people we know that mental health is not as good as in general the population, and then add on more [like] being a black person, or an immigrant, or and the add on HIV, like it’s always adding on […] (P2).

As a concrete example, some participants wanted more peer support opportunities that would target young people in particular.

There were some young people at [the patient organization]. But most of them were older. I was invited to some kind of event for younger people where they kind of got together in [a major city in Sweden]. But I was in that meeting and I liked that because it was a group of younger people and that I miss. That, I would have changed. (P4)

It is important to note that participants also mentioned that peer support should be constantly evolving to meet the evolving needs of PLHIV.

Lastly, participants perceived having the right location as another key facet of a successful peer support experience. Among participants, formal forms of peer support were provided through patient organizations. They also perceived infectious disease clinics as a safe space. Some even viewed them as a suitable location for peer support to be provided because they are a place that all PLHIV in Sweden frequent.

I never imagined that it would have been the NGOs [providing peer support]. I thought that was up to the clinics to do that. I thought that was the service of the clinics, like that would have been the clinics for me (P2).

Some participants perceived and experienced online platforms as a safe location to begin speaking with PLHIV before meeting with others in real life. They mentioned using a variety of online platforms such as dating applications, websites, web forums specifically for PLHIV, and mainstream social media applications. Through these platforms, participants were able to have varying degrees of contact with other PLHIV. Some chose to participate through only reading what others had written on the platforms, while others engaged through publicly posting on group pages, individual pages, and web forum threads. Others engaged in one-on-one contact with other PLHIV through private messaging and emailing. One reason why participants chose to engage in peer support via online platforms was because it allowed them to practice forming connections with other PLHIV while also still maintaining some form of anonymity. Additionally, participants also used online platforms because it allowed them to connect with PLHIV around the world.

I started to go into these dating sites online, and talking to people online, and in other countries. People in Russia, some people in Belgium, some people in Denmark, Norway. Maybe it was a little bit to like to protect myself, and to just like protect myself like through doing these actions […] (P4).

This was especially the case with PLHIV residing in rural areas, where there were very few other PLHIV in the nearby vicinity.

It’s super quiet, and you don’t meet people. It’s a very quiet town, and no one to talk to. So, I usually do my conversation online. I go to dating apps [and] when I meet people, that’s where I try to ask [about their thoughts on HIV] quickly … and if I know the mentality’s right, I [talk to that person more] (P10).

It is important to note that how participants found their way peer support varied. After diagnosis, some participants were referred to peer support services at patient organizations by a clinic professional, while others were left to seek both informal and formal support services on their own. For participants referred to patient organizations, this referral was done at the discretion of clinic professionals.

## Discussion

Our findings showed that participants both perceived and experienced peer support as a necessity for recently diagnosed individuals. To our knowledge, this study is the first of its kind within a Swedish context.

From our findings it can be inferred that formal referral processes for newly diagnosed PLHIV to peer support services are limited. Participants experienced either being referred to peer support services by a clinic professional or were left to navigate this process on their own. In Sweden, peer support could be housed within, or provided in partnership with infectious disease clinics. This informal referral pathway within infectious disease clinics is noteworthy and suggests a system-wide gap in services to meet the quality of life needs of PLHIV. As such, further research is needed to understand how the accessibility of peer support services may differ among PLHIV in Sweden. It is important to note that not having standardized referral pathways to peer support services for recently diagnosed PLHIV is not unique to Sweden. This practice has also been similarly observed in Australian clinical settings as well [32, 33].

Moreover, participants perceived peer support as something that should be tailored to meet a person’s unique demographic needs. Among participants, peer support was experienced as not solely focusing on acquiring knowledge about HIV, but also on how HIV intersected with other areas of life such as dating, employment, and identity. Those who acquired HIV in their late teens and early twenties experienced peer support organizations consisting of primarily older men and desired spaces and activities specifically for youth. The importance of providing tailored peer support has been observed in other studies [7, 34–37]. Within a Swedish context however, our finding regarding the unmet peer support needs of youth is novel and warrants further investigation.

Furthermore, the results of our study add to existing literature by showing how peer support within Sweden helps recently diagnosed men navigate challenges such as stigma management. According to various quality of life studies, PLHIV encounter stigma, financial stress, mental health issues, and loneliness at higher rates than PNLHIV [5, 19, 22]. Our study results build on this and illustrate that peer support helps participants develop stigma management skills and provides them with a social network that can help ameliorate challenges relating to mental health and loneliness. Within our study, many participants perceived public knowledge about HIV to be low and a contributing factor to the stigma they faced. Studies have shown that even though HIV in Sweden is not as stigmatized as it once was, a lack of knowledge among the general public is thought to be a contributing factor to existing stigmatizing [38, 39]. According to Mehdiyar et al. [40], within Sweden little research has been done on potential differences in the experience of stigma among PLHIV of Swedish background, and PLHIV of immigrant background. The results of our study add to the literature in this area by showing that although both Swedish and immigrant PLHIV used peer support for stigma management, their fears about stigmatization varied. This finding suggests that peer support in Sweden should be tailored to address stigma uniquely faced by PLHIV of diverse backgrounds.

Finally, our findings provide new insights about the nature of peer support relationships. In their study, Peterson et al. [1] found that peer support could occur within embedded social networks via family, partners, and friends. They also found that peer support should be made available beyond created social networks, because embedded social networks could yield different effects on health and wellbeing for PLHIV when compared to less personal sources of support. Our study adds to these findings by showing that in the U = U era, embedded social networks seem to have extended beyond PLHIV. We found that people not living with HIV but with proximity to HIV were occasionally perceived as peers by participants. This finding implies that among participants, peers can be defined as individuals connecting over shared commonalities. This shared connection can be solely due to the shared commonality of having an HIV positive status, can be the result of other shared commonalities in addition to having an HIV positive status, or can be due to having shared commonalities irrespective of HIV status. We speculate this may be the result of PLHIV perceiving HIV as a lifelong chronic illness, which subsequentially may broaden their views on who might be regarded as a peer.

Our study’s definition of a peer and peer support mirrored the definitions used by others who have previously studied the peer support needs of PLHIV. However, our results indicate that more research is needed to understand the criteria for being a peer during the U = U era. Our findings suggest that existing conceptualizations of what constitutes peer support for PLHIV might be too narrow, because a key criterion for being a peer is having an HIV positive status [1, 8, 9]. This could be in part due to these definitions being developed prior to this era. Moreover, Peterson et al. [1] speculated that having HIV was a key component of PLHIV developing friendships with one another. They also asserted that peers held a special significance as support providers because they understood things that those without HIV were unable to [1]. Our findings support this speculation. Among some participants, connections formed during peer support encounters developed into friendships. These friendships originated in the comfort of having a shared understanding of what it is like to live with HIV. These friendships eventually grew to encompass other shared commonalities outside of having a shared HIV status.

### Methodological considerations

In our study, a few limitations are present. Because all participants are under 50 years of age, study results may not be applicable to men 50 years of age and older. Moreover, because this study focuses on the experiences of recently diagnosed men, study results may not be applicable to the needs and experiences of recently diagnosed women. Additionally, because we defined recently diagnosed as living with HIV for no more than ten years, there was a risk for recall bias among respondents. Attempts to minimize this were made by having a clearly defined study aim, clearly formulated research questions, and developing an interview guide that facilitated rich conversation. Lastly, even though sufficient information power had been reached via rich descriptions of what it was like receiving and providing peer support, we cannot exclude that additional participants would have contributed additional perspectives.

This study has many strengths as well. The analytical process allowed all members of the research team to assess analytical decisions, increasing the confirmability of study results. Additionally, engaging in conversations with a partner patient organization for PLHIV during the study design process enriched and informed the research team’s approach to this study’s design. Moreover, by situating this study within U = U era, our results provide insight into the current peer support needs of men adjusting to life with HIV within a low prevalence, high ART adherence setting. As a result, these results may be transferable to other low HIV prevalence, high ART adherence contexts where similarities are present.

## Conclusion

In the U = U era, men adjusting to life with HIV in Sweden perceived peer support as assisting with acquiring the skills needed to live successfully with the virus and managing the challenges of HIV related stigma and status disclosure. Further research is needed on the differences in support needs between younger men and older men living with HIV in Sweden. Additionally, further research is needed to assess the accessibility of peer support services in Sweden. Moreover, study results suggest that conceptualizations of peer support for PLHIV within current literature may be too narrow, indicating that further research is needed on how the criteria for being a peer may have changed within this era.
