# Correction: Finding a safe space for learning and exploration: A qualitative study of recently diagnosed men’s experiences of peer support for HIV in Sweden

**DOI:** 10.1371/journal.pone.0319035

**Published:** 2025-02-05

**Authors:** The PLOS One Staff

## Notice of republication

This article was republished on January 28, 2025, to correct an error in the author list: Arielle N’Diaye was erroneously displayed as Arielle N’ Diaye. The publisher apologizes for the error. Please download this article again to view the correct version. The originally published, uncorrected article and the republished, corrected article are provided here for reference.

## Supporting information

S1 File (PDF)

S2 File (PDF)
